# Relevance of *KCNJ5* in Pathologies of Heart Disease

**DOI:** 10.3390/ijms241310849

**Published:** 2023-06-29

**Authors:** Karisa M. Meyer, Nipun Malhotra, Jung seo Kwak, Mona El Refaey

**Affiliations:** 1 Department of Surgery, Division of Cardiac Surgery, The Ohio State University, Columbus, OH 43210, USA; meyer.1320@osu.edu (K.M.M.); nipun.malhotra@osumc.edu (N.M.); kwak.114@osu.edu (J.s.K.); 2 The Dorothy M. Davis Heart and Lung Research Institute, The Ohio State University, Columbus, OH 43210, USA

**Keywords:** *KCNJ5*, atrial fibrillation, sinus node dysfunction, long QT, familial hyperaldosteronism

## Abstract

Abnormalities in G-protein-gated inwardly rectifying potassium (GIRK) channels have been implicated in diseased states of the cardiovascular system; however, the role of GIRK4 (Kir3.4) in cardiac physiology and pathophysiology has yet to be completely understood. Within the heart, the K_ACh_ channel, consisting of two GIRK1 and two GIRK4 subunits, plays a major role in modulating the parasympathetic nervous system’s influence on cardiac physiology. Being that GIRK4 is necessary for the functional K_ACh_ channel, *KCNJ5*, which encodes GIRK4, it presents as a therapeutic target for cardiovascular pathology. Human variants in *KCNJ5* have been identified in familial hyperaldosteronism type III, long QT syndrome, atrial fibrillation, and sinus node dysfunction. Here, we explore the relevance of *KCNJ5* in each of these diseases. Further, we address the limitations and complexities of discerning the role of *KCNJ5* in cardiovascular pathophysiology, as identical human variants of *KCNJ5* have been identified in several diseases with overlapping pathophysiology.

## 1. Introduction

The G-protein inwardly rectifying potassium channel subunit 4 (GIRK4) is primarily expressed in the heart [1], pancreas [2], and adrenal glands, particularly the zona glomerulosa [3]. Within the heart, the cardiac muscarinic K^+^ channel (K_ACh_), formed of both G-protein inwardly rectifying potassium channel subunits 1 and 4 (GIRK1 and GIRK4), plays a major role in modulating the parasympathetic influence of cardiac activity [1]. Interestingly, GIRK4-deficient mice, which completely lacked the K_ACh_ channels, displayed resting heart rates similar to controls yet showed decreased heart rate variability and a diminished bradycardic response to parasympathetic stimulation or after adenosine administration [4]. In addition, GIRK4-deficient mice demonstrated reduced cholinergic regulation of sinoatrial node pacemaker activity, as well as delayed restoration of resting heart rate after sympathetic stimulation [5]. This further highlights the crucial role of the GIRK4 subunit in the proper function of the K_ACh_ channel and, in turn, in heart rate regulation. The K_ACh_ channel displays the greatest expression in the atria, the sinoatrial node, and the atrioventricular node, with minor expression in ventricular myocytes [6]. However, mouse models demonstrated that the ventricular K_ACh_ channel does not appear to have a significant role in the regulation of heart rate and heart rate variability [6]. Moreover, tissue-specific GIRK channel genetic ablation demonstrated that atrial GIRK channels are primarily responsible for the effects of the parasympathetic nervous system's influence on cardiac physiology [7].

The activation and deactivation of the K_ACh_ channel rely majorly on G-protein signaling and regulators of G-protein signaling (RGS) proteins, respectively. First, acetylcholine released from the vagus nerve binds to the associated cardiac muscarinic (M2) receptor. When activated in the presence of acetylcholine, these receptors slow the heart rate, demonstrating negative dromotropic effects and little to no inotropic effects [8]. The bound M2 receptor incorporates the GDP-bound G-protein trimer (αβγ) to form a G-protein-coupled receptor, GPCR-Gα (GDP) complex, and concurrently, the activated GPCR exchanges GDP for GTP, releasing Gα (GTP) and Gβγ subunits from the complex. The Gβγ protein dimer binds to and activates the K_ACh_ channel, allowing the flow of K^+^ ions. This results in hyperpolarization of the cardiac action potential, decreasing atrial and ventricular myocyte activity and further slowing the heart rate. Hydrolysis of Gα (GTP) to Gα (GDP) via RGS causes the dissociation of Gβγ from the channel and reformation of the Gα (GDP) by complex to stop the signaling pathway [9]. In addition to these mechanisms, GIRK channel activity was also shown to be modulated by G-protein independent signaling pathway, i.e., cholesterol and ethanol activate GIRK with the help of phosphatidylinositol-4, 5-biphosphate (PIP2) [10,11]. It has been suggested that PIP2 might induce the structural changes in GIRK channels that are necessary for the binding of regulatory substances (including Gαβγ proteins) [12].

**Figure 1:**
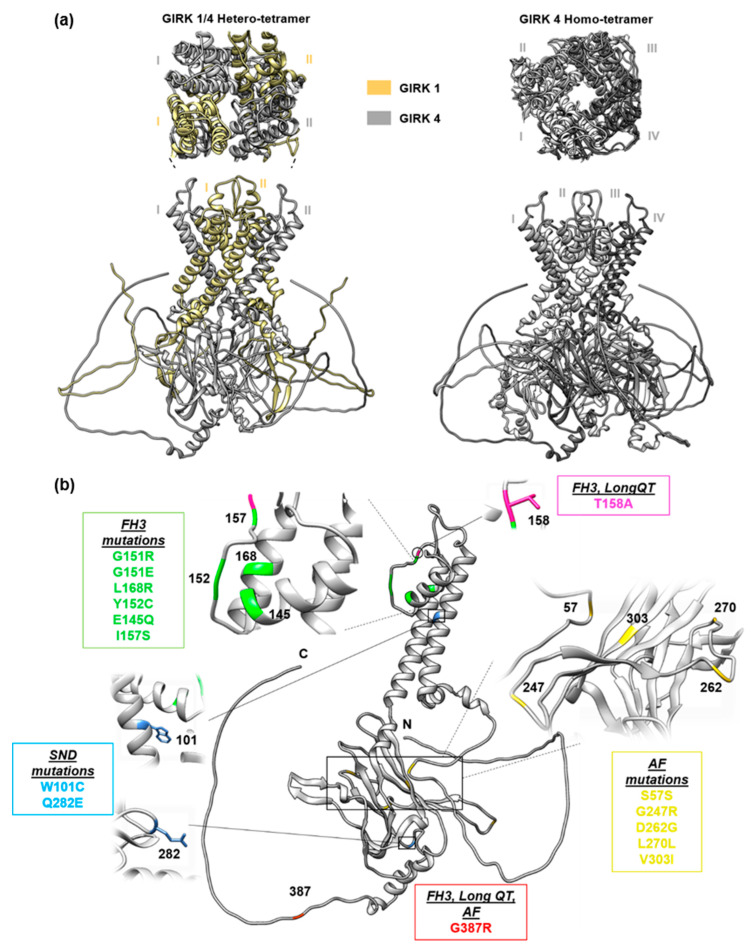
(**a**) Structural representation of functional hetero-tetrameric and homo-tetrameric GIRK channels. Hetero-tetramer is composed of two GIRK1 (Tan, I–II) and two GIRK4 subunits (Grey, I–II), whereas homo-tetramer is composed of four GIRK4 subunits (Grey, I–IV). (**b**) Location of *KCNJ5* variants identified in familial hyperaldosteronism type III (G151R, G151E, T158A, L168R, Y152C, E145Q, I157S, G387R), atrial fibrillation (S57S, G247R, D262G, L270L, V303I, G387R), sinoatrial node dysfunction (Q282E, W101C), and long QT syndrome (T158A, G387R). GIRK1 and GIRK4 PDBs were sourced from the Alpha Fold Database (https://alphafold.ebi.ac.uk/, accessed on 27 April 2023) and illustrations were made using UCSF Chimera (https://www.cgl.ucsf.edu/chimera/, accessed on 27 April 2023).

Abnormalities in G-protein-gated inwardly rectifying potassium (GIRK) channels have been previously implicated in diseased states of the cardiovascular system; however, the complex role of GIRK4 (Kir3.4) in cardiac physiology and pathophysiology has yet to be completely understood [13]. Encoded for by the *KCNJ5* gene, GIRK4 is an inwardly rectifying potassium channel subunit which can exist either with GIRK1 in a GIRK1/4 hetero-tetramer (composed of two GIRK1 and two GIRK4 subunits) or on its own as a homo-tetramer of four GIRK4 subunits (GIRK4 homo-tetramer) [14] (Figure 1). Referred to as the native K_ACh_ channel, the GIRK1/GIRK4 hetero-tetrameric inwardly rectifying potassium channel consists of four subunits oriented about a central pore with four-fold symmetry [14]. GIRK4 is necessary for the proper processing and localization of GIRK1 in a functional hetero-tetramer, as GIRK1 is unable to properly localize to the cell membrane without association with either GIRK2 or GIRK4 [1,15,16].

The functional GIRK4 monomer is 419 amino acids long, whereas GIRK1 (encoded by the *KCNJ3* gene) is slightly longer, with 501 amino acids [17]. Each GIRK subunit contains an N-terminal intracellular region, two transmembrane alpha-helix, an inner helix and an outer helix, a pore region, and a C-terminal intracellular region. Previous findings involving truncated and chimeric subunits highlighted the importance of the C terminus in the GIRK function [15,18,19], particularly that the GIRK4 C terminal is essential for cell surface localization of a functional channel, whereas the GIRK1 C terminal promotes retention inside the cell in the absence of GIRK4 [15,18]. Specifically, GIRK4 amino acids (350–374) are indispensable for cell surface localization of GIRK4 homo-tetramers and GIRK1/GIRK4 hetero-tetramers, whereas amino acids (375–399) are essential for functional GIRK1/4 hetero-tetramers [15].

Parasympathetic response, through modulation of the current produced by the K_ACh,_ (*I*_KACh_), is generally considered pro-fibrillatory in the atrium, whereas it is anti-fibrillatory in the ventricles. In contrast, the sympathetic response is pro-fibrillatory for both the atrium and the ventricles [20]. In addition to being pro-fibrillatory in the atrium, excessive parasympathetic control can disrupt normal heart physiology to cause effects such as decreased heart rate and increased heart rate variability [21]. Meanwhile, lack of parasympathetic control has been linked with decreased heart rate variability and diminished heart recovery after sympathetic stimulation, thereby increasing the risk of sudden cardiac death [20]. Therefore, given the necessity of GIRK4 in functional the K_ACh_ channel, as well as the role of this channel in modulating parasympathetic influence on heart rate and rhythm, the *KCNJ5* gene poses as a promising therapeutic target for diseased states of the cardiovascular system. This review will discuss the role of inherited *KCNJ5* variants in familial hyperaldosteronism type III and long QT syndrome and explore the relevance of *KCNJ5* in both atrial fibrillation (AF) and sinus node dysfunction (SND). Further, we address the complexities of discerning the true role of *KCNJ5* in cardiac physiology and pathophysiology and pose suggestions for future directions.

## 2. Familial Hyperaldosteronism Type III

Congenital hyperaldosteronism caused by variation in the *KCNJ5* gene is classified as familial hyperaldosteronism type III [22]. Disease-causing variants diminish the selectivity of GIRK4-containing inward-rectifying potassium channels in the adrenal gland [3]. These less-selective mutant channels permit the flow of other ions, including Na^+^, thus altering membrane potential and opening voltage-gated Ca^2+^ ion channels [23]. The subsequent inward flow of Ca^2+^ ions activates the aldosterone biosynthetic pathway [22]. Patients with familial hyperaldosteronism type III often present with polyuria, polydipsia, hypokalemia, and treatment-resistant hypertension [22]. Furthermore, these patients are at increased risk for adverse cardiovascular effects, as aldosterone has been implicated in the pathogenesis of numerous cardiac abnormalities, including heart failure (HF) and arrhythmias [24,25].

Genomic analysis of 22 patients with aldosterone-producing adenoma (APA) revealed an L168R *KCNJ5* variant in 6 patients, as well as a G151R *KCNJ5* variant in 2 patients [3]. A later study suggested these two are the most common primary hyperaldosteronism disease-causing variants [26]. When expressed in HEK293T cells, both *KCNJ3/KCNJ5*^L168R^ and *KCNJ3/KCNJ5*^G151R^ mutant channels demonstrated depolarization and loss of ion selectivity [3]. Located within the second transmembrane domain, the side chain of residue 168 points into the selectivity filter; therefore, the substitution of leucine with arginine could alter protein structure in such a way that the selectivity filter is affected [3] (Figure 1).

An additional investigation consisting of ten individuals from four different families, all with primary aldosteronism and early-onset hypertension, revealed that all ten individuals had mutations in *KCNJ5* which affect the G151 amino acid located within the selectivity filter of the inwardly rectifying potassium channel [27] (Figure 1). Two of the families displayed a G151R variant, which has been previously identified in patients with APAs [3,27]. The other two families displayed a novel G151E variant [27]. Patients varied phenotypically depending on which variant they had. Those with the G151R variant were not responsive to spironolactone treatment and required eventual appendectomy, whereas those with the G151E variant did respond to spironolactone treatment. Further, only patients with the G151R variant displayed disease progression with age [27]. When transfected in 293T cells, the G151E variant resulted in great cell lethality, which improved in a low Na^+^ medium, suggesting lethality could be the result of excess Na^+^ conductance through the mutant channel. Further, such cell lethality could explain why, unlike G151R, the G151E mutation has not been seen in primary hyperaldosteronism patients with APAs [27].

Another familial study, consisting of a father and two children with primary hyperaldosteronism [28], revealed a heterozygous T158A mutation co-segregated with the disease [3]. The T158A variant occurs just above the selectivity filter of the inwardly rectifying potassium channel (Figure 1). The substitution of hydrophilic threonine with hydrophobic alanine disrupts hydrogen bonds, altering channel structure and reducing selectivity [3]. When the T158A variant was transfected into 293T cells, mutant channels displayed reduced ion selectivity and membrane depolarization [3].

Three additional variants, Y152C, E145Q, and I157S, have been identified in case studies of patients with primary hyperaldosteronism [29,30,31]. Transfection of the Y152C variant into HAC15 cells (HAC15 is an epithelial-like cell line that was clonally isolated from the adrenal gland of a carcinoma patient) produced a mutant channel displaying electrophysiological properties similar to those observed in other disease-causing mutations, including increased Na^+^ permeability and membrane depolarization [3,29]. In HAC15 cells overexpressing the Y152C mutation, there was an up-regulation of both *CYP11B2* and its transcription factor *NR4A2* [29]. The E145Q variant, which was previously observed in two APAs [32], disrupts the formation of an essential salt bridge to produce a mutant channel with loss of ion selectivity and inward rectification [33] (Figure 1). When transfected in NCI-H295R cells (human adenocarcinoma cell line), mutant channels displayed larger inward and outward currents, as well as Na^+^-dependent depolarization [30]. Further, HAC15 cells expressing the mutant channel displayed increased intracellular Ca^2+^ as well as up-regulation of *CYP11B2* and *NR4A2* [30]. The I157S variant occurs within a hydrophobic pocket at the C-terminal, away from the selectivity filter (Figure 1). The substitution of hydrophobic isoleucine with hydrophilic serine results in unfavorable conformational changes, which disrupt the selectivity filter of the channel [31].

During genomic analysis, Cheuh et al. identified a G387R variant in 6 of 223 APA patients with mutations in *KCNJ5* [26]. When transfected in HEK293T cells, the G387R mutant channel displayed a similar current–voltage relationship to the WT channels and did not alter the ion selectivity of the channel [26]. Further, an in vitro study using HAC15 cells demonstrated that the G387R mutation did not increase aldosterone production [26]. Taken together, these results suggest that G387R is not a primary aldosteronism-causing variant. However, there are limitations to the model systems used in this study. While often used as a model for aldosterone production, HAC15 cells are not as hyperpolarized nor excitable as zona glomerulosa cells in the adrenal glands [34]. Therefore, it is possible that the G387R variant does affect aldosterone production but that it was not accurately replicated in cellular studies.

## 3. *KCNJ5* in Long QT Syndrome

Long QT syndrome (LQTS) is a potentially life-threatening arrhythmic condition characterized by delayed myocardial repolarization that causes QT prolongation and increased risk for torsades de pointes (TdP), syncope, and even sudden cardiac death (SCD) [35]. A subtype of long QT syndrome (long QT 7), Andersen–Tawil syndrome is a rare genetic disease predominantly caused by pathogenic variants in the *KCNJ2* gene, which encodes for Kir2.1 [36]. However, in 2014, G387R, and T158A, *KCNJ5* variants were implicated in Anderson–Tawil syndrome [37]. Kokunai et al. propose that Kir2.1 and Kir3.4 form a functional hetero-tetramer, which is disrupted by the G387R variant [37]. Injection of G387R Kir3.4 and wild-type Kir2.1 into oocytes produced a significant reduction in inwardly rectifying potassium currents compared to injection of wild-type Kir3.4 and wild-type Kir2.1 [37]. Moreover, the identified G387R variant is within the C-terminus of the Kir3.4 protein, within the region that was proven essential for forming the Kir3.4/Kir3.1 hetero-tetramer [15] (Figure 1).

The G387R *KCNJ5* variant identified in Andersen–Tawil syndrome was also identified in an additional subtype of long QT syndrome, Romano–Ward syndrome (long QT 13) [38,39]. The G387R *KCNJ5* variant resulted in a loss of function of the K_ACh_ channel. Further, when the G387R variant was co-expressed with wild-type GIRK1 in HEK293 cells, reduced GIRK1 and GIRK4 were observed at the plasma membrane and in cytoplasmic fractions. Therefore, the G387R *KCNJ5* variant likely interferes with the formation of the functional GIRK1/GIRK4 K_ACh_ channel, which is essential for the repolarization of the cardiac action potential [39].

In contrast to the causative role of *KCNJ5* in long QT syndrome that is suggested by the aforementioned studies, an international analysis of the 17 genes reported as causative for long QT syndrome found limited evidence to support a causal role of *KCNJ5* variation in long QT syndrome [40]. Given the conflicting evidence, further investigation is necessary to establish the role (or lack thereof) of *KCNJ5* in long QT syndrome.

## 4. *KCNJ5* in Atrial Fibrillation

Being that excessive parasympathetic influence can be pro-fibrillatory in the atrium, and the atrial K_ACh_ channel is crucial for mediating parasympathetic influence on cardiovascular physiology, *KCNJ5*/GIRK4 present as an interesting target in the pathogenesis of AF [7,20]. When abolished via GIRK4 knockout, mice lacking *I*_KACh_ were demonstrated to be resistant to carbachol-induced AF. In contrast, carbachol administration induced AF in 10 out of 14 WT mice [41]. At baseline, GIRK4 KO mice displayed shorter sinus cycle lengths and longer ventricular effective refractory periods compared to WT controls. After carbachol administration, GIRK4 KO mice demonstrated lower changes from baseline in sinus node recovery time, as well as reduced changes in the atrioventricular interval [41]. Such findings suggest that inhibition or abolishment of *I*_KACh_ could be preventative against AF, thereby implicating a therapeutic role of K_ACh_ channel inhibitors in the treatment of AF.

When *I*_K1_ (the cardiac inwardly rectifying potassium current) [42] and *I*_KACh_ were measured in isolated atrial myocytes from sinus rhythm (SR) controls and patients with chronic AF (cAF), *I*_K1_ was elevated, and *I*_KACh_ was reduced in cAF patients [43]. Further, patients with cAF displayed action-potential duration (APD) shortening, more negative resting membrane potential, and attenuated response to muscarinic stimulation [43]. These observations align with the subsequent finding that cAF patients display drug-resistant, receptor-independent constitutive *I*_KACh_ activity [44]. Like the previous, this study also utilized right atrial appendages from patients with SR controls and patients with cAF. Patients with cAF displayed higher basal current. Using tertiapin, constitutive active *I*_KACh_ was suggested to contribute to elevated basal current in cAF [44]. Single-channel analysis of K_ACh_ in cAF revealed that spontaneous channel openings were resistant to block via the muscarinic receptor antagonist atropine [44].

When electrophysiological properties of the K_ACh_ channel were studied using left and right atrial appendages from SR controls, patients with paroxysmal AF (pAF), and cAF patients, pAF patients demonstrated a left-to-right basal current gradient which was not observed in cAF patients [45]. Immunoblotting revealed reduced expression of both GIRK1 and GIRK4 in RA of pAF and cAF patients. No such reduction was present in LA tissue. SR controls demonstrated a significant right-to-left gradient in atrial GIRK1 and GIRK4 protein expression [45]. Accordingly, carbachol administration in SR controls produced *I*_KACh_ with a greater current in RA than in LA [45]. Taken together, these findings suggest a right-to-left *I*_KACh_ current gradient in SR, which is disrupted in AF conditions [45]. An additional study concluded that heterogeneous atrial expression of GIRK4 could underlie the molecular mechanisms of adenosine-induced AF [46]. Intravenous adenosine administration has been demonstrated to induce atrial arrhythmia; however, the mechanisms of this arrhythmogenesis were previously uncertain [47]. Using isolated human atria, Li et al. demonstrated that despite similar average-action potential duration (APD) at baseline, adenosine administration induced heterogeneous APD shortening which was greater in RA than LA. Adenosine-induced APD shortening was reversed in the presence of tertiapin, indicating a GIRK channel-dependent mechanism [46].

Genetic analysis of unrelated patients with sporadic AF revealed several heterozygous variants in the *KCNJ5* gene with potential relevance for genetic predisposition to AF: *KCNJ5* c.785A>G, p.D262G, *KCNJ5* c.907G>A, p.V303I, and *KCNJ5* c.1159G>A, p.G387R [48]. The G387R variant has been previously identified and reported as a heterozygous-dominant variant in a Han Chinese family suffering from type 13 long QT syndrome, a ventricular repolarization disorder [38,39]. The D262G and V303I variants were predicted to be disease-causing, D262G specifically causing significant changes during functional analysis, displaying increased current compared to wild-type channels. Structural modeling indicated that residue D262 is located within the intracellular region of the GIRK protein complex [48] (Figure 1). Within the GIRK protein complex, the intracellular domain plays an important role in modulating channel activity, as it is the site of binding for activating G-proteins [49]. Therefore, the D262G variant may interfere with the normal regulation of the channel to alter its activity. Yamada et al. suggest that the D262G variant contributes to the pathogenesis of AF via atrial repolarization heterogeneity, which has been previously attributed to AF [48].

Another study identified a GIRK4p.G247R variant in a patient with a single episode of AF. This variant was not seen in any other AF or control patients; however, the proband’s son was heterozygous for the same variant. Unlike his mother, who was also heterozygous, the son never displayed an arrhythmogenic phenotype [50]. Using Xenopus laevis oocytes, GIRK4p.G247R/GIRK1 and GIRK4/GIRK1 were expressed in a 1:1 ratio to replicate the effects of a heterozygous variant. Oocytes expressing the G247R mutation had average currents that were significantly reduced compared to oocytes only expressing wild-type GIRK1 and GIRK4 [50]. When GIRK4p.G247R was expressed alone or only in the presence of GIRK4, mutant homo-tetrameric channels displayed even greater current reduction than wild-type homo-tetrameric GIRK4 channels [50]. When co-expressed in oocytes with the muscarinic acetylcholine type 2 receptor, mutant homo-tetrameric channels demonstrated a significant reduction of acetylcholine-induced signaling in the mutant channel [50]. The G247R mutation occurs at the region just downstream of the GIRK4 region, which has been implicated in G-protein interactions; therefore, it is likely that the substitution of glycine with the larger arginine residue disrupts normal GIRK channel activation [51] (Figure 1).

Two additional single-nucleotide polymorphisms (SNPs) of *KCNJ5* c.171C>T (rs6590357) and c.810G>T (rs7118824), have been independently associated with early-onset lone AF in both Han Chinese and Caucasian populations [52,53]. The variants have been associated with action potential duration shortening and reduction of the effective refractory period, both of which have been implicated in the development and maintenance of AF [54,55].

## 5. *KCNJ5* in Sinus Node Dysfunction

GIRK/K_ACh_ channels play an important role in negatively regulating sinoatrial node (SAN) pacemaker activity under parasympathetic stimulation [14]. Therefore, GIRK proteins, particularly GIRK4, have the potential to play a role in the pathophysiology of sinus node dysfunction (SND). SND is a heterogenous disorder characterized by abnormal cardiac impulse generation and can be further classified as either primary or secondary SND depending on pathogenesis [56,57]. Primary SND can be attributed to genetic factors, whereas secondary SND is the resultant effect of another heart pathology such as HF, AF, cardiac ischemia, etc. [57,58,59].

Studies have suggested the potential relevance of GIRK4 and *I*_KACh_ in the pathophysiology of primary sinus node dysfunction [60,61,62]. Ablation of functional K_ACh_ channel via GIRK4 knockout was able to rescue SAN bradycardia and associated arrhythmia in mouse models of SND [62]. These mutant mice, expressing a dominant negative non-conductive HCN4 subunit, lacked functional *I*_f_ and demonstrated phenotypes consistent with those of patients with SND [62]. Mesirca et al. suggest that these results evidence *I*_f_ and *I*_KACh_ as counterbalancing currents [62]. Similarly, symptoms of SND such as bradycardia and heart block were abolished by both genetic inactivation and pharmacological targeting of K_ACh_ in additional mouse models with genetic ablation of L-type Ca_v_1.3 Ca^2+^ channels (Ca_v_1.3^−/−^) and T-type Ca_v_3.1 Ca^2+^ channels (Ca_v_3.1^−/−^), as well as deficiency of the Na^+^ channel Na_v_1.5 (Na_v_1.5^+/−^) [60,61]. Moreover, the capacity of *I*_KACh_ suppression to improve heart rate reinforces its potential as a target in SND [60,61,62].

*KCNJ5* was also demonstrated to play a role in the pathogenesis of secondary SND [63,64]. Genetic ablation of K_ACh_ via GIRK4 knockout was preventative against training-induced bradycardia [64]. Further, GIRK4 knockout mice did not demonstrate the same exercise-induced downregulation of *I*_f_ (pacemaker current or funny current important in sinoatrial node automaticity) [65]_,_ *I*_CaT_ (current produced by voltage-gated T-type Ca^2+^ channels) [66,67], and *I*_CaL_ (current produced by voltage-gated L-type Ca^2+^ channels) [66,68,69] as WT mice [64]. Additionally, pharmacological blockers of K_ACh_ were shown to prevent conduction abnormalities in human SAN maintained ex vivo [63]. Serving as a model of HF, one of many cardiac pathologies which can serve as a precursor to secondary SND, this study revealed that precise targeting of *I*_KACh_ could improve dysfunction of the sinoatrial node without affecting other cardiac physiology [63].

Recently, a gain of function of the K_ACh_ channel was seen in familial SND via a variant in the *GNB2* gene. This gene encodes for the beta2 subunit of the heterotrimeric G-protein complex involved in the signaling pathway of the K_ACh_ channel [70]. In a study done by Kuß et al. [71], analysis of 52 unrelated patients with idiopathic SND resulted in the identification of a novel non-synonymous variant in *KCNJ5*, where a hydrophobic, non-polar tryptophan at position 101 is substituted with cysteine, a hydrophilic, polar amino acid (Figure 1). This tryptophan residue has orthologous and paralogous conservation, thereby making this change particularly harmful [71]. In this study, co-expression of GIRK1 with an equal ratio of wild-type and W101C mutated GIRK4 resulted in a stronger K_ACh_ channel current with partial loss of inward rectification. When co-expression was done with only W101C mutated GIRK4, i.e., without wild-type GIRK4, it had an even more pronounced gain-of-function [71]. The resultant elevated efflux of K^+^ ions can lead to hyperpolarization of the sinoatrial node, thereby altering SAN pacemaking and contributing towards bradycardia. Interestingly, this gain-of-function variant, *KCNJ5* p.W101C is localized in the first transmembrane domain of Kir3.4 towards the cytosol and results in the alteration of the spermidine binding site without affecting the ion selectivity. Hence the reduced inward rectification of K_ACh_ channels with mutant GIRK4p.W101C contributes to SND through impaired spermidine binding [71,72,73].

## 6. Complexities of *KCNJ5* in Cardiovascular Disease

**Figure 2:**
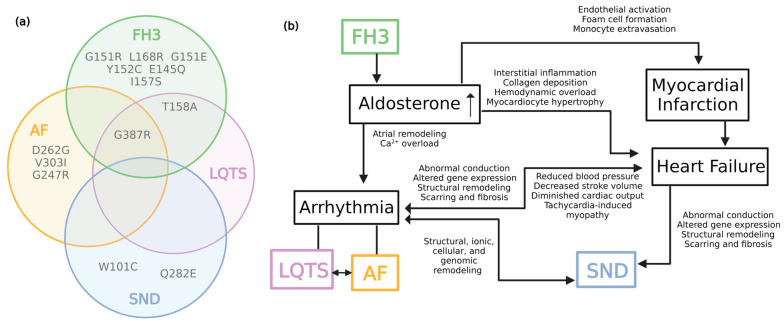
(**a**) Schematic illustration of *KCNJ5* missense variants which have been identified in familial hyperaldosteronism type III (FH3), long QT syndrome (LQTS), sinoatrial node dysfunction (SND), and atrial fibrillation (AF) [3,26,27,28,29,30,31,37,48,50,71]. (**b**) Visual representation of the complex interplay between familial hyperaldosteronism type III (FH3), long QT syndrome (LQTS), sinoatrial node dysfunction (SND), and atrial fibrillation (AF) [25,58,59]. (Created with BioRender.com, https://app.biorender.com/illustrations/6449326e9927dfab781afd6b, created on 27 April 2023).

Great complexity arises when trying to discern the role of *KCNJ5* in cardiovascular disease. As discussed, numerous studies have implicated *KCNJ5* in the pathophysiology of several conditions. However, cardiac phenotypes overlap across these conditions [25,58,59], imposing the following questions: Is each disease truly distinct, with *KCNJ5* having a role in the pathophysiology of each? Or rather, could *KCNJ5* directly contribute to the pathophysiology of one disease, the effects of which in turn promote other pathogeneses? (Figure 2).

Familial type III hyperaldosteronism is caused by *KCNJ5* variants which disrupt ion channel selectivity, resulting in excess aldosterone synthesis, which can contribute to cardiac pathology [22]. Specifically, patients with primary hyperaldosteronism are at increased risk of AF [74] (Figure 2). While evidence has suggested a role for *KCNJ5* in the pathophysiology of AF, it could be possible that *KCNJ5* variants contribute to the pathogenesis of AF as a result of cardiovascular changes due to primary hyperaldosteronism.

In addition to increasing the risk of arrhythmia, aldosterone can contribute to HF [24]. HF and AF are both conditions that contribute to the pathogenesis of secondary SND [57] (Figure 2). While studies have associated *KCNJ5* variants with SND, it is important to distinguish whether *KCNJ5* is contributing to the pathology of primary SND or rather contributing to another condition which in turn promotes secondary SND. A study by Holmegard et al. contradicts the direct involvement of K_ACh_ channels in SND pathogenesis [75]. In this study, genes encoding K_ACh_ channel subunits, i.e., *KCNJ3* and *KCNJ5*, from 43 SND patients (Danish population, <60 years) with pacemaker implantation and no structural impairment, were sequenced [75]. Four previously known genetic variations in *KCNJ5* were identified. Three of the variants were synonymous, S57S (c.171C>T, rs6590357), L270L (c.810G>T, rs7118824), H278H (c.834T>C, rs7118833, while the non-synonymous variant), and Q282E (c.844C>G, rs7102584), predicted to be benign [75]. Two of these variants, c.171C>T and c.810G>T, have been associated with the initiation and maintenance of lone early-onset AF [53,54]. Therefore, findings suggest that genetic variants in *KCNJ5* do not contribute to the pathogenesis of SND; rather, SND combined with AF is a genetic disorder.

## 7. Future Implications

There is great evidence implicating the *KCNJ5* gene and its encoded protein GIRK4 in the pathophysiology of diseased states of the cardiovascular system. The next steps for advancing this understanding include the development of novel model systems for studying the role of GIRK4 in various pathways. The majority of the models used thus far are knockout mouse models, which, while helpful for establishing the role of the protein of interest, do not necessarily replicate what is occurring in patients. Numerous variants in *KCNJ5* have been previously identified in patient populations; to truly understand what is occurring in pathology, it is essential to replicate these specific variants in model systems. Mouse models demonstrating point mutations have been developed using CRISPR/Cas9 [76]. Animal models are not the only option; induced pluripotent stem cells (iPSCs) have been used for studying cardiovascular disease [77]. Because they are derived from patient tissues, iPSCs can serve as accurate models for studying what is occurring in a diseased state [78]. Further, iPSCs have been effectively differentiated into both atrial and ventricular myocytes [79]. Given that GIRK4 is primarily expressed in atrial myocytes, iPSCs can be used to study the role of *KCNJ5* in cardiovascular disease. However, it is necessary to note that iPSCs provide an in vitro, rather than in vivo, model of diseased states. Therefore, iPSCs should be used in combination with other model systems for an adequate understanding of what is occurring in diseased states.

In addition to their use in disease modeling, iPSCs can also be used for drug screening [78]. This could be advantageous in the development of novel treatments for cardiac pathologies, as several modulators of GIRK channels have been identified, suggesting their potential to be utilized and developed into novel medications for both AF and SND [80]. Notable compounds include VU0458554 and benzopyran-G1. VU0468554 is an inhibitor that displayed selectivity for cardiac GIRK1/GIRK4 tetramers, while benzopyran-G1 is an inhibitor that targets GIRK1-containing channels, including K_ACh_ [81,82]. An additional inhibitor selective for K_ACh_, XAF-1407, effectively terminated arrhythmic phenotypes in equine and goat models of AF [83,84]. Moreover, tertiapin, a peptide extracted from bee venom, selectively inhibits the K_ACh_ channel [85]. Tertiapin was demonstrated to effectively terminate AF in canine models [86]. Further, inhibition of K_ACh_ using tertiapin was able to improve dysfunction of the sinoatrial node in mice lacking the L-type Ca_v_1.3 Ca^2+^ channels (Ca_v_1.3^−/−^), mice lacking the T-type Ca_v_3.1 Ca^2+^ channels (Ca_v_3.1^−/−^), and mice haplo-insufficient for the Na^+^ channel Na_v_1.5 (Na_v_1.5^+/−^) [60,61].

Additionally, evidence suggests that gene therapy could be an effective method for targeting abnormalities of GIRK4 associated with diseased states. A study found that small hairpin RNA (shRNA) was effective at silencing GIRK4 in human atrial myocytes [87]. The review by Cao et al. provides a comprehensive insight into the technicalities and mechanisms of gene therapy for cardiovascular disease [88]. The application of gene therapy to cardiovascular disease will allow not only increased treatment options but allow treatment to be better personalized per the needs of individual patients.

A crucial caveat when discussing GIRK4 as a therapeutic target in diseased states of the cardiovascular system is the necessity for developed treatments to be cardio-specific. While GIRK4 is primarily expressed in cardiac tissues, its structure is similar to that of other GIRK subunits with crucial functions in other tissues. GIRK1, GIRK2, and GIRK3 are expressed in the brain and, like GIRK4, form tetramers [89]. Because of the similarities among the various GIRK subunits, it is important to ensure that any therapy targeting GIRK4 in the cardiovascular system does not disrupt the function of GIRK channels in other tissues.

An additional note to consider when discussing the role of *KCNJ5* in pathologies of heart rate regulation and arrhythmogenesis is the difficulty of replicating the complexities of the human genome in models of disease. Typical animal and mouse models are monogenic, focusing on the role of one gene in diseased states; however, this might not be what is occurring in patients. These diseases may be oligogenic, with phenotypes relying not only on interactions between numerous genes but also being influenced by the interactions of these genes with the environment. Therefore, it is important to consider the complex diversity of the genome and the impact of environmental factors when investigating diseases of the cardiovascular system.

In summary, *KCNJ5* plays an important, albeit not completely understood, role in cardiovascular pathology. Therefore, it presents a promising therapeutic target. However, further research dissecting its exact role in abnormalities of the cardiovascular system is a must; both novel animal models (replicating patient variants) and iPSCs can be used to accomplish this feat. Additionally, both pharmacological interventions and gene therapy targeting GIRK4/*KCNJ5* have been demonstrated to reverse cardiovascular abnormalities in model systems and should be further refined with the hopes of use in clinical settings.
